# Effectiveness of interventions to reduce ordering of thyroid function tests: a systematic review

**DOI:** 10.1136/bmjopen-2015-010065

**Published:** 2016-06-03

**Authors:** Zhivko Zhelev, Rebecca Abbott, Morwenna Rogers, Simon Fleming, Anthea Patterson, William Trevor Hamilton, Janet Heaton, Jo Thompson Coon, Bijay Vaidya, Christopher Hyde

**Affiliations:** 1 NIHR CLAHRC South West Peninsula, University of Exeter Medical School, University of Exeter, Exeter, Devon, UK; 2 Clinical Chemistry, Royal Cornwall Hospital, Treliske, Truro, UK; 3 Department of Endocrinology, Royal Devon and Exeter Hospital, Exeter, UK; 4 Exeter Test Group, Institute of Health Research, University of Exeter Medical School, University of Exeter, Exeter, UK

**Keywords:** thyroid function tests, test ordering, behaviour changing interventions, systematic review, medical tests

## Abstract

**Objectives:**

To evaluate the effectiveness of behaviour changing interventions targeting ordering of thyroid function tests.

**Design:**

Systematic review.

**Data sources:**

MEDLINE, EMBASE and the Cochrane Database up to May 2015.

**Eligibility criteria for selecting studies:**

We included studies evaluating the effectiveness of behaviour change interventions aiming to reduce ordering of thyroid function tests. Randomised controlled trials (RCTs), non-randomised controlled studies and before and after studies were included. There were no language restrictions.

**Study appraisal and synthesis methods:**

2 reviewers independently screened all records identified by the electronic searches and reviewed the full text of any deemed potentially relevant. Study details were extracted from the included papers and their methodological quality assessed independently using a validated tool. Disagreements were resolved through discussion and arbitration by a third reviewer. Meta-analysis was not used.

**Results:**

27 studies (28 papers) were included. They evaluated a range of interventions including guidelines/protocols, changes to funding policy, education, decision aids, reminders and audit/feedback; often intervention types were combined. The most common outcome measured was the rate of test ordering, but the effect on appropriateness, test ordering patterns and cost were also measured. 4 studies were RCTs. The majority of the studies were of poor or moderate methodological quality. The interventions were variable and poorly reported. Only 4 studies reported unsuccessful interventions but there was no clear pattern to link effect and intervention type or other characteristics.

**Conclusions:**

The results suggest that behaviour change interventions are effective particularly in reducing the volume of thyroid function tests. However, due to the poor methodological quality and reporting of the studies, the likely presence of publication bias and the questionable relevance of some interventions to current day practice, we are unable to draw strong conclusions or recommend the implementation of specific intervention types. Further research is thus justified.

**Trial registration number:**

CRD42014006192.

**Strengths and limitations of this study**

- The current systematic review was conducted following the methods recommended by the Cochrane Collaboration. We worked to a prespecified protocol and consider our findings to be robust.
- This is the first review focusing specifically on the effectiveness of interventions designed to reduce unnecessary ordering of thyroid function tests.
- The evidence suggests that, in general, such interventions are effective in reducing the volume, changing the pattern of ordering, improving compliance with guidelines or reducing the cost of thyroid function tests ordered. Whether such changes reflect more appropriate test ordering remains unclear as measures of appropriateness were rarely reported.
- However, the poor quality of evidence, the significant heterogeneity in study design and the likely presence of publication bias and selective reporting did not allow strong conclusions and more specific recommendations to be made and precluded pooling the result from the individual studies.

## Introduction

Thyroid dysfunctions including hypothyroidism and hyperthyroidism are among the most common medical conditions with prevalence 3.82% (3.77–3.86%) and incidence 259.12 (254.39–263.9) cases per 100 000/year in Europe.^1^ Both undertreatment and overtreatment of these conditions may have serious consequences for the patient's health and, therefore, correct and timely diagnosis and monitoring are important.^2^ ^3^

The diagnosis of thyroid dysfunctions, however, is challenging as they present with common and non-specific symptoms: a range of laboratory investigations, such as thyroid-stimulating hormone (TSH), free thyroxine (FT4) and free tri-iodothyronine (FT3) are readily available to rule them in or out. In the UK alone 10 million thyroid function tests (TFTs) are ordered each year at an estimated cost of £30 million.^4^

Although national guidelines for the use of TFTs exist,^4^ a recent audit of general practitioners' (GPs) ordering patterns conducted by our group in the South West of England found that there is a sixfold variation in the rates of test requests between different practices. The study also demonstrated that only about 24% of this variation could be accounted for by variation in the prevalence of hypothyroidism and socioeconomic deprivation.^5^ *The National Health Service (NHS) Atlas of Variation in Diagnostic Services* published in November 2013^6^ reported even more extreme variation in the annual rate of TFTs ordered by GPs per practice population across different primary care trusts in England. In this report, the estimated annual rate for TSH ordered by GPs ranged from 6.2 to 355.8 per 1000 practice population (57-fold variation). The reported numbers for FT4 and FT3 were 14.6–231.1 (16-fold) and 0.42–17.0 (40-fold) per 1000 practice population, respectively (p. 122).

A qualitative study we conducted identified a wide range of mechanisms that might be responsible for the variation, including the presence of inappropriate test ordering.^7^ Given the continuous rise of thyroid test requests,^8^ ^9^ which is disproportionate to the increase in the incidence and prevalence of thyroid conditions,^9^ and the fact that these investigations make up a significant proportion of all laboratory tests ordered in primary care,^10^ there is a need to help clinicians avoid inappropriate thyroid testing. Such testing not only increases laboratory workload and wastes scarce resources but may also have a negative impact on patients' health through further unnecessary tests and inappropriate treatment.^11^

The effectiveness of interventions designed to reduce the number of unnecessary medical tests has already been evaluated in a number of systematic reviews.^12–16^ Owing to their broad scope, however, the results are too general and of little help when it comes to designing interventions that target specific test ordering behaviour. The effect of the same intervention may vary considerably across different tests, even when they belong to the same diagnostic modality.^10^ ^17–19^ We conducted a systematic review investigating the effect of behavioural interventions on the ordering of TFTs. We believed a more narrowly focused approach with respect to target behaviour might produce more applicable results and thus better inform the development and implementation of interventions specifically designed to improve TFT ordering.

## Methods

In conducting the review, we followed the recommendations of the Cochrane Collaboration.^20^ MEDLINE, EMBASE and the Cochrane Database of Systematic Reviews were searched using a predefined search strategy (see online supplementary appendix 1). The original search covered the period up until November 2013 and was updated on 1 May 2015. Also, the bibliographies of the included studies and other relevant publications were scrutinised for additional articles. Studies were selected independently by two reviewers (ZZ and RA) with all disagreements resolved through discussion and, if necessary, arbitration by a third reviewer (CH or BV). In the first round, all electronically identified citations were screened at title and abstract level. Full-text copies of potentially relevant articles were retrieved for full-text screening. Studies were included in the review if they met the following prespecified criteria:

- Evaluated the effectiveness of interventions designed to reduce the number of inappropriately ordered TFTs (regardless of whether they were the only targeted tests or not).
- Were randomised controlled trials (RCTs), non-randomised controlled studies or single-group before and after studies (including both those with trend before and after and those with just one time point before and after).
- The outcomes were one or more of the following: change in the total number of TFTs, the number of inappropriately ordered tests, the test-related expenditure or health benefits to individual patients (eg, the number of unnecessary tests or treatments avoided).
- Reported the specific effect that the intervention had on the targeted TFTs.

10.1136/bmjopen-2015-010065.supp1 Supplementary appendix 1

Studies that targeted TFTs along with other tests and reported only the average effect (across all tests) were excluded. We included all studies that used the rate of inappropriately ordered TFTs as an outcome measure regardless of the definitions they used. Appropriateness of test ordering is usually judged against local protocols or guidelines that may vary from place to place or change over time. We accepted all definitions even when they were outdated or did not fit in with the current UK guidelines. We did not use the setting and the targeted clinicians' characteristics as inclusion criteria but explored, as far as possible, their potential impact on the study outcomes. The methodological quality of the included studies was assessed independently by ZZ and RA using the Effective Public Health Practice Project tool which allows the assessment of all study designs with the same rubric.^21^ The method of synthesis was narrative; meta-analysis was not used because of the anticipated clinical heterogeneity, particularly in terms of the interventions. The framework for the analysis was based on an existing typology of behaviour change intervention types:^12^ ^15^

- Educational interventions;
- Guideline and protocol development and implementation;
- Changes to funding policy;
- Reminders of existing guidelines and protocols;
- Decision-making tools, including test request forms and computer-based decision support;
- Audit and feedback.

All work conformed with a protocol defined and published ahead of the review being started (PROSPERO, registration number CRD42014006192).

## Results

**Figure 1:**
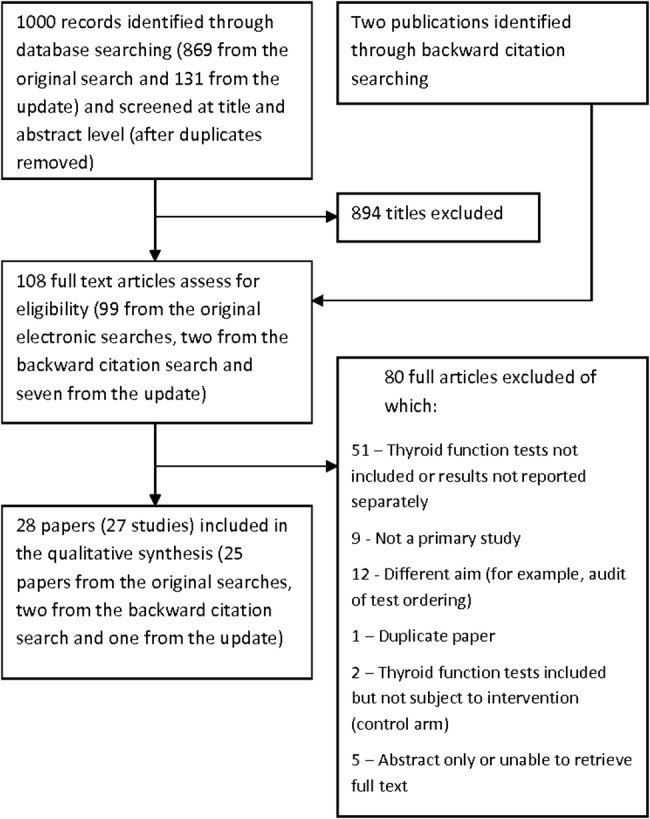
Flow chart of the selection process.

The initial electronic searches produced 1282 hits of which, after removing duplicates, 869 were screened at title and abstract level and 99 were selected for full-text screening. Twenty five of these papers, with two additional papers identified through backward citation searching, met our prespecified criteria, and were included in the review.^10^ ^17–19^ ^22–44^ The update search identified another 131 records of which, after screening the titles and abstracts, 7 were selected for full-text screening and 1 met the inclusion criteria.^45^ It should be noted that two papers^46^ ^47^ were excluded because in these studies TFTs were allocated to the control arm and, therefore, were not affected by the interventions. Thus, the total number of papers included in the review was 28 of which 2 reported on the same study, the second reporting a long-term follow-up.^30^ ^31^ The selection process and the reasons for full-text exclusion are detailed in figure 1.

### Study characteristics

The characteristics of the included studies are summarised in table 1 and the evaluated interventions are presented in table 2. Ten studies were conducted in the USA, six in the UK and the rest in Australia (n=3), France (n=3), Canada (n=2), the Netherlands (n=1), Sweden (n=1) and New Zealand (n=1). All studies were published in English, except for one in Dutch, which was partly translated by a native Dutch speaker with a background in healthcare research.^38^ The papers were published between 1979^43^ and 2014:^45^ seven of them were published before 1991, nine between 1991 and 2000, and 12 after 2000. Fourteen studies were conducted in a hospital setting including general and psychiatric hospitals, medical assessment units, emergency departments and a supraregional liver unit, with the remainder in primary care or community settings (table 1).

**Table 1:** Characteristics of the included studies

Study and country	Study design	Setting	Targeted test users	Target tests	Thyroid tests
Adlan *et al*,^22^ UK	Before and after; single site	Medical assessment unit (acutely ill hospital patients)	Physicians	TFTs only	TSH, FT4, FT3, TPOAb, TRAb
Baker *et al*,^23^ UK	Cluster RCT	GP practices	GPs, locums, GPs in training and nurses	5 frequently ordered laboratory tests suspected of being inappropriately ordered	TSH and FT4
Berwick and Coltin,^17^ USA	Controlled cross-over; 3 sites	Ambulatory centres at health maintenance organisation	Internists and adult nurse practitioners	13 laboratory and imaging tests suspected of being excessively ordered	TT4
Chu *et al*,^24^ Australia	Before and after; single site	Adult tertiary referral teaching hospital ED	Interns and residents	Frequently ordered blood tests suspected of being excessively ordered	TFTs unspecified
Cipullo and Mostoufizadeh,^19^ USA	Before and after; single site	Community hospital	Medical staff (unspecified)	A range of high-volume laboratory procedures	TFTs unspecified but change in TT3 rate used as a measure of impact
Daucourt *et al*,^25^ France	Cluster RCT	General and psychiatric hospitals	Physicians	TFTs only	TSH, FT4, FT3, TRH test
Dowling *et al*,^26^ USA	Before and after; single site	Innercity community health centre	Family practice residents	TSHs only (complete blood count with differential used as a comparator)	TSH
Emerson and Emerson,^27^ USA	Before and after; single site	University medical centre	Residents	All laboratory tests	TSH, FT3, FT4, TT3, TT4 (individual and cascade)
Feldkamp and Carey,^28^ USA	Before and after; single site	Metropolitan hospital and 22 satellite clinics (inpatients and outpatients)	Physicians	TFTs only	TSH, TT3, TT4
Gama *et al*,^18^ UK	Controlled study; single site	District general hospital (inpatients and outpatients)	General medicine physicians	All laboratory tests	TFTs unspecified
Grivell *et al*,^29^ Australia	Before and after; single site	Tertiary care community hospital	Consultants	55 most commonly requested laboratory tests or test groups	TT4
Hardwick *et al*,^44^ Canada	Before and after; multiple sites	All non-hospital-based laboratories in British Columbia	All users of non-hospital-based laboratories	TFTs only	TT3, TT4
Horn *et al*,^45^ USA	Interrupted time series with a parallel control group; multiple sites	Alliance of 5 multispecialty group practices	Primary care physicians	27 laboratory tests	TSH
Larsson *et al*,^30^ Mindemark and Larsson^31^ (follow-up), Sweden	Before and after; multiple sites	Primary healthcare centres	GPs and laboratory technicians	Various laboratory tests	TSH, FT4, TT4, TT3
Nightingale *et al*,^32^ UK	Before and after; single site	Supraregional liver unit at teaching hospital	House officers	Various laboratory tests	TSH
Rhyne and Gehlbach,^43^ USA	Before and after; single site	Family medicine centre	Physicians	TFTs only	Thyroid function panel including TT4 and TT3
Schectman *et al*,^33^ USA	Controlled study; single site	Primary care health maintenance organisation	Physicians, physician assistants and nurse practitioners	TFTs only	TSH, TT4, TT3
Stuart *et al*,^34^ Australia	Before and after; single site	Urban public hospital ED	Consultants, registrars, junior medical officers and casual medical staff	All laboratory tests	TFTs unspecified
Thomas *et al*,^10^ UK	Cluster RCT	Primary care practices in 1 NHS covered area	Family practitioners	9 laboratory tests suspected of being inappropriately ordered	TSH
Tierney *et al*,^35^ USA	RCT; single site	Academic general medicine practice	Physicians (residents and faculty)	8 commonly ordered diagnostic tests	TSH
Tomlin*et al*,^36^ New Zealand	Controlled study; multiple sites	New Zealand primary care	All GPs on the New Zealand Medical Council's register compared with locum GPs and other medical specialists	TFTs only (but related programmes targeted inflammatory response tests and tests for infectious diarrhoea)	TSH, FT3, FT4
Toubert *et al*,^37^ France	Before and after; single site	Teaching hospital	Physicians (various specialties, including endocrinologists)	TFTs only	TSH, FT3, FT4, TPOAb, TRAb, TgAb
van Gend *et al*,^38^ The Netherlands	Before and after; multiple sites	GP practices in 1 geographical area	GPs	15 laboratory tests	TSH, FT4
van Walraven *et al*,^39^ Canada	Retrospective interrupted time series; multiple sites	All private non-hospital-based laboratories in Ontario	Physicians ordering tests from non-hospital laboratories	7 laboratory tests; 6 unaffected tests were chosen as controls	TSH, TT4, TT3
Vidal-Trécan *et al*,^40^ France	Before and after; multiple sites	A network of 50 non-profit university hospitals in the Paris region	Physicians	TFTs only	TSH, TT4, TT3, FT3, FT4
Willis and Datta,^41^ UK	Before and after; single site	Medical admissions unit at a district general hospital	Nurses	Three potentially inappropriately requested test sets: thyroid profile, lipid profile and coagulation screen	Thyroid profile (unspecified)
Wong *et al*,^42^ USA	Controlled study; single site	University teaching hospital	Physicians	TFTs, creatinine kinase and lactate dehydrogenase isoenzyme	TSH, TT4, TT3

ED, emergency department; FT3, free tri-iodothyronine; FT4, free thyroxine; GP, general practitioner; NHS, National Health Service; RCT, randomised controlled trial; TFTs, thyroid function tests; TgAb, thyroglobin antibody; TPOAb, thyroid peroxidase antibody; TRAb, thyrotropin receptor antibody; TRH, thyrotropin-releasing hormone test; TSH, thyroid-stimulating hormone; TT3, total tri-iodothyronine; TT4, total thyroxin.

**Table 2:** Characteristics of the included studies: interventions

Study and country	Setting	Educational programmes	Guidelines and protocols	Changes to funding	Reminders	Decision tools	Audit and feedback
Single-mechanism interventions							
Adlan *et al*,^22^ UK	Hospital		X				
Berwick and Coltin,^17^ USA*	Primary care	X					XX
Chu *et al*,^24^ Australia	Hospital		X				
Cipullo and Mostoufizadeh,^19^ USA	Hospital		X				
Daucourt *et al*,^25^ France*	Hospital				X	X	
Emerson and Emerson,^27^ USA	Primary care					X	
Feldkamp and Carey,^28^ USA	Hospital		X				
Gama *et al*,^18^ UK	Hospital						X
Grivell *et al*,^29^ Australia	Hospital						X
Horn *et al*,^45^ USA	Primary care					X	
Larsson *et al*,^30^ Mindemark and Larsson^31^ (follow-up), Sweden	Primary care	X					
Schectman *et al*,^33^ USA	Primary care	X					
Tierney *et al*,^35^ USA	Primary care					X	
Thomas *et al*,^10^ UK*	Primary care				X		
Multifaceted interventions							
Baker *et al*,^23^ UK	Primary care		X				X
Daucourt *et al*,^25^ France*	Hospital				X	X	
Dowling *et al*,^26^ USA	Primary care	X					X
Hardwick *et al*,^44^ Canada	Primary care		X	X			
Nightingale *et al*,^32^ UK	Hospital	X				X	X
Rhyne and Gehlbach,^43^ USA	Primary care	X	X				
Schectman *et al*,^33^ USA*	Primary care	X			X		X
Stuart *et al*,^34^ Australia	Hospital	X	X				X
Thomas *et al*,^10^ UK*	Primary care				X		X
Tomlin *et al*,^36^ New Zealand	Primary care	X	X				X
Toubert *et al*,^37^ France	Hospital		X		X		
van Gend *et al*,^38^ The Netherlands	Primary care					X	X
van Walraven *et al*,^39^ Canada	Primary care		X	X		X	
Vidal-Trécan *et al*,^40^ France	Hospital	X	X			X	
Willis and Datta,^41^ UK	Hospital	X	X				
Wong *et al*,^42^ USA	Hospital		X			X	

XX—two independent interventions of the same type.

*Study comparing directly two alternative interventions (Schectman *et al*^33^ had a non-comparative single-intervention design in its first phase and a multifaceted comparative design in the second phase; Thomas *et al*^10^ compared a single-mechanism (reminders) vs multifaceted (feedback plus reminders) interventions; Daucourt *et al*^25^ compared two single-mechanism interventions with their combination and usual practice defined as simple diffusion of guidelines).

Education, guidelines/protocols and audit/feedback were the most common types of intervention employed. Reminders and decision tools were less commonly used and changes to funding were assessed in only two studies (table 2). Only three studies reported evaluation of computer-based test ordering, two of which were quite old, published in 1988^35^ and 1994,^32^ respectively. The recent one^45^ evaluated only a limited aspect of computerised test ordering—the display of costs of tests being ordered.

The median duration of the interventions was 12 months (IQR 6–12 months, range 2 days to 36 months) and four studies examined test ordering after the intervention had ended.^26^ ^30^ ^31^ ^33^ ^35^ Description of the interventions was usually limited and often insufficient to allow replication. Where detail was provided, it revealed significant variability in the design and implementation of interventions superficially belonging to the same category. For instance, educational interventions varied in terms of content, intensity and frequency, method of delivery, who delivered and who received the intervention as well as other characteristics which are likely to affect their effectiveness and appropriateness for different contexts and purposes. Most interventions were targeted at both senior and junior doctors. In four studies, only junior doctors were included;^24^ ^26^ ^27^ ^32^ four studies included other medical staff, such as nurses, physician assistants and laboratory technicians,^17^ ^23^ ^30^ ^33^ and in one study, the intervention was specifically directed at nurses^41^ (table 1).

In terms of targeted tests, 10 studies focused exclusively on TFTs^22^ ^25^ ^26^ ^28^ ^33^ ^36^ ^37^ ^40^ ^43^ ^44^ while the rest targeted either a selection of laboratory tests suspected of being overutilised or had a wider scope including imaging as well as laboratory investigations. Of the targeted TFTs, five studies focused exclusively on TSH,^10^ ^26^ ^32^ ^35^ ^45^ two on TSH and FT4,^23^ ^38^ four reported an average result without specifying the individual tests^18^ ^24^ ^34^ ^41^ and the remaining studies targeted other combinations (table 1). As the studies spanned a long period of time, different generations of tests were used and the guidelines against which the appropriateness of test ordering was judged varied. However, in most studies, the recommended testing strategy was based on TSH as a single first-line test for suspected thyroid dysfunction and for monitoring patients on thyroid replacement hormones.

The effectiveness of the evaluated interventions with respect to TFTs is summarised in table 3 with additional information provided in table 4. The effect of the interventions was measured using a range of outcomes. Thus, 22 studies measured changes in the volume of test ordering expressed either as the absolute number of tests ordered for a period of time or normalised by the number of registered patients, visits or a similar parameter. To capture the effect on the pattern of test ordering, seven studies reported separate results for different TFTs^27^ ^28^ ^36^ ^37^ ^40^ ^42^ ^44^ and three studies measured the change in the ratios of two different tests (for instance, whether the ratio ‘TSH:all TFTs’ has increased as a result of new guidelines recommending TSH as a single first-line test).^30^ ^31^ ^36^ ^38^ More direct evaluation of the appropriateness of testing was carried out by measuring adherence to protocols or guidelines^25^ ^26^ ^32^ ^33^ ^37^ ^43^ with two of these studies reporting underutilisation as well as overutilisation.^25^ ^26^ Five studies reported effectiveness in terms of expenditure^34–36^ ^39^ ^44^ and one study reported an estimate of the number of tests avoided as a result of the intervention.^39^ In one study, researchers made an effort to investigate whether the evaluated intervention (in this case, a test-ordering protocol) had had any adverse effects on patient outcomes by conducting an audit of 4000 case notes and concluded that “No adverse patient outcomes relating to underutilisation of investigations attributable to the protocol were identified.” (ref. 34, p. 133).

**Table 3:** Summary of results: effectiveness of the interventions with respect to thyroid function tests

Outcome	Study	Intervention		Direction	Large effect	Statistically significant change	RCT design	Notes on outcome measures
Single-mechanism interventions								
Test numbers or rates	Adlan *et al*^22^	Guidelines		+	+	+	−	Per cent admissions offered TFTs
	Berwick and Coltin^17^	Education (test specific)		+	−	NR	−	Per 100 encounters
	Berwick and Coltin^17^	Feedback on cost		+	−	NR	−	Per 100 encounters
	Berwick and Coltin^17^	Feedback on yield		−	+	NR	−	Per 100 encounters
	Chu *et al*^24^	Guidelines		+	+	+	−	Per 100 ED visits
	Cipullo and Mostoufizadeh^19^	Guidelines		+	−	NR	−	Per discharge
	Gama *et al*^18^	Feedback		+	+	+	−	Per outpatient visit
	Emerson and Emerson^27^	Request form redesign		+	−	+	−	
	Feldkamp and Carey^28^	Guidelines		+	−	NR	−	Per 1000 patients
	Grivell *et al*^29^	Feedback		−	+	NR	−	
	Horn *et al*^45^	Display of cost of tests being ordered		+	−	−	−	Per 1000 visits
	Schectman *et al*^33^	Educational memorandum		+	−	+	−	Per patient
	Thomas *et al*^10^	Feedback		+	−	+	+	Per 10 000 registered patients
	Thomas *et al*^10^	Reminders		+	−	+	+	Per 10 000 registered patients
Appropriateness (compliance)	Daucourt *et al*^25^	Pocket memory card		+	−	−	+	Proportion of TFTs ordered in accordance with the guidelines
	Daucourt *et al*^25^	Request form redesign		+	+	+	+	Proportion of TFTs ordered in accordance with the guidelines
	Schectman *et al*^33^	Educational memorandum		+	+	+	−	Compliance with TSH-only strategy
	Schectman *et al*^33^	Reminders		+	−	+	−	Compliance with TSH-only strategy
Expenditure	Tierney *et al*^35^	Display of computer- generated probability estimates		+	−	−	+	Per visit
CV	Berwick and Coltin^17^	Education (test specific)		−	−	NR	−	
	Berwick and Coltin^17^	Feedback on cost		+	+	NR	−	
	Berwick and Coltin^17^	Feedback on yield		+	+	NR	−	
Pattern	Emerson and Emerson^27^	Request form redesign		+	+	NR	−	Sought to shift to TSH and thyroid cascade
	Feldkamp and Carey	Guidelines		+	+	NR	−	Sought to shift to TSH and TSH-based algorithm
	Larsson *et al*^28^ and Mindemark and Larsson^31^	Years 1–2	Education	+	−	+	−	Sought to shift to TSH; and reduce ordering of TT3 and FT4 relative to TSH. Summary based on TSH/all TFTs ratios
	Larsson *et al*^28^ and Mindemark and Larsson^31^	Years 2–6	Education	−	−	+	−	Sought to shift to TSH; and reduce ordering of TT3 and FT4 relative to TSH. Summary based on TSH/all TFTs ratios
Multiple-mechanism interventions								
Test numbers or rates	Baker *et al*	Education and guidelines		+	−	−	+	Per 1000 registered patients
	Daucourt *et al*	Pocket memory card and request form redesign		+	−	+	+	Proportion of TFTs ordered in accordance with the guidelines
	Dowling *et al*^26^	Education and feedback		+	−	+	−	Per patient visit
	Hardwick *et al*^44^	Funding policy and guidelines		+	−	NR	−	
	Rhyne and Gehlbach^43^	Education and guidelines		+	+	+	−	Per 100 encounters
	Schectman *et al*	Feedback and reminders		+	−	+	−	Per patient
	Thomas *et al*^10^	Feedback and reminders		+	−	+	+	Per 10 000 registered patients
	Tomlin *et al*^36^	Education and feedback and guidelines		+	+	+	−	Per year per GP
	Toubert *et al*^37^	Guidelines and reminders		+	+	NR	−	
	van Walraven *et al*^39^	Guidelines and funding policy		+	+	+	−	Summary based on decrease in the proportion of TT4 and T3RU
	van Walraven *et al*^39^	Guidelines and request form redesign		+	−	+	−	Summary based on decrease in TSH utilisation
	Vidal-Trécan *et al*^40^	Education and guidelines and request form redesign		+	−	NR	−	Summary based on the total number of TFTs
	Willis and Datta^41^	Education and guidelines		+	+	+	−	Per admission
	Wong *et al*^42^	Guidelines and request form redesign		+	+	NR	−	
Appropriateness (compliance)	Dowling *et al*^26^	Education and feedback		+	+	+	−	Per cent TSH indicated
	Nightingale *et al*^32^	Education and feedback and protocol management system		+	+	NR	−	Per cent patients requiring a particular investigation according to protocol who were actually tested
	Rhyne and Gehlbach^43^	Education and guidelines		+	−	−	−	Per cent ‘high’ and ‘low’ indications
	Schectman *et al*^33^	Feedback and reminders		+	−	−	−	
	Toubert *et al*^37^	Guidelines and reminders		+	+	+	−	Per cent appropriate
Expenditure	Hardwick *et al*^44^	Funding policy and guidelines		+	+	NR	−	
	Stuart *et al*^34^	Education and feedback and guidelines		+	+	+	−	
	Tomlin *et al*^36^	Education and feedback and guidelines		+	−	NR	−	
Pattern	Hardwick *et al*^44^	Funding policy and guidelines		+	−	NR	−	Sought to decrease proportion of TT3 as TFTs requested. Summary based on per cent of TFTs TT3
	Tomlin *et al*^36^	Education and feedback and guidelines		+	+	+	−	Sought to shift to TSH. Summary based on per cent TFTs TSH alone
	Toubert *et al*^37^	Guidelines and reminders		+	+	NR	−	Sought to shift to TSH. Summary based on per cent TFTs TSH alone
	van Gend *et al*^38^	Feedback and test form redesign		+	+	NR	−	Sought to shift away from TT4. Summary based on FT4:TSH ratio
	Vidal-Trecan *et al*^40^	Education and guidelines and request form redesign		+	−	NR	−	Sought to shift to TSH. Summary based on the proportion of FT3 and TSH
	Wong *et al*^42^	Guidelines and request form redesign		+	+	NR	−	Sought to decrease ordering of ‘complete’ thyroid panel to more selective use of individual tests

*Direction*: ‘+’ indicates result favours behaviour change intervention; ‘−’ opposite. *Large effect*: ‘+’ indicates risk difference is ≥20%; ‘−’ <20%. Statistically significant change: ‘+’ indicates 95% CI do not include no effect or p<0.05; ‘−’ opposite. RCT *design*: ‘+’ indicates study design is RCT; ‘−’ not RCT.

CV, coefficient of variation; ED, emergency department; FT3, free tri-iodothyronine; FT4, free thyroxine; GP, general practitioner; NR, indicates item not reported; RCT, randomised controlled trial; T3RU, tri-iodothyronine resin uptake; TFTs, thyroid function tests; TSH, thyroid-stimulating hormone; TT3, total tri-iodothyronine; TT4, total thyroxin.

**Table 4:** Summary of results: additional information

Study and country	N at baseline	Intervention and outcome measure	Level at baseline	Level postintervention		Effect on outcome
Randomised controlled study designs						
Baker *et al*^23^ UK	Practices: 17 (I) 16 (C)	*Intervention(s)*: G+FB *Measure(s)*: median (IQR) number of TFTs per 1000 registered patients	17.4 (8.0, 39.5) (I) 22.7 (10.4, 30.9) (C)	19.8 (6.2, 42.3) (I) at 6 months 19.5 (10.3, 31.1) (C) at 6 months 17.7 (6.6, 43.3) (I) at 9 months 17.3 (10.1, 34.0) (C) at 9 months 13.2 (6.3, 35.7) (I) at 12 months 20.9 (13.3, 35.3) (C) at 12 months		For TFTs, the difference in mean change in test rate from baseline to 4th quarter was—1.45 in favour of the I (95% CI −4.59 to 1.68) but was not statistically significant (p=0.35) The intervention had no significant effect on the other tests, too.
Daucourt *et al*,^25^ France	Hospital wards: 17 (PMC+TRF) 20 (TRF) 17 (MPC) 13 (C)	*Intervention(s)*: PMC, TRF, PMC+TRF *Measure(s)*: GCR	NA	77.9% (95% CI 68.9% to 87.0%) (MPC+TRF) 82.6% (95% CI 73.1% to 92.1%) (TRF) 73.4% (95% CI 56.7% to 90.1%) (MPC) 62.0% (95% CI 47.7% to 76.4%) (C)		GCR was significantly higher in PMC+TRF compared with the C (OR 2.65; 95% CI 1.52 to 4.62; p<0.01); slightly lower compared with TRF and slightly higher compared with MPC but the differences were not statistically significant No difference between MPC and the C (OR 1.28; 95% CI 0.75 to 2.19; p=0.37)
Thomas *et al*,^10^ UK	Practices: 21 (FB+R) 22 (FB) 22 (R) 20 (C)	*Intervention(s)*: FB, R, FB+R *Measure(s)*: median (IQR) TFT requests per 10 000 patients per practice	750 (515–1329) (C) 829 (476–1412) (FB) 961 (476–1338) (R) 891 (392–1277) (FB+R)	795 (552–1466) (C) 802 (432–1359) (FB) 891 (490–1250) (R) 800 (287–1077) (FB+R)		Is were significantly less likely to request TFTs (FB group: OR 0.90 (0.84 to 0.97), p=0.005; R group: OR 0.82 (0.83–0.95), p=0.001). Across all targeted tests, intervention practices were significantly less likely to order tests.
Tierney *et al*,^35^ USA	Scheduled visits: 7658 (I) 7590 (C)	*Intervention(s)*: display of computer-generated probability estimates *Measure(s)*: charges per scheduled visit (in USA$)	NA	1.25 (C) 1.12 (I)		TSH showed 10.3% decrease in charges per visit in the I group but this difference was not significant at p=0.05. Across all targeted tests, there was a significant reduction in charges per visit (−8.8%, p<0.05), with return to baseline at 3 months follow-up.
Non-randomised controlled study designs						
Berwick and Coltin,^17^ USA	35 internists and 30 adult nurses at 3 centres (total number of visits not given)	*Intervention(s)*: TSE, FBC and FBY *Measure(s)*: per cent change in the number of TT4 ordered per 100 encounters and the CV of test ordering rates	TT4 tests per 100 encounters: Site X: 72 Site Y: 72 Site Z: 45 Range of rates of TT4 use: Site X: 40–273 Site Y: 36–122 Site Z: 35–78	*Change*:	*CV*:	TT4 use declined in the TSE and FBC groups but increased in FBY; CV decreased in FBC and FBY but increased in the C group and showed very small increase in TSE group. The statistical significance of these results is NR. Across all tests statistically significant decline in test ordering (14.2%, p=0.012) was observed only in the FBC group.
				C: +1.7%	+15.7%	
				TSE: −15.9%	+0.5%	
				FBC: −12.1%	−23.6	
				FBY: +34.0%	−28.2	
Gama *et al*,^18^ UK	Outpatient visits: 2991 (I) 4393 (C)	*Intervention(s)*: FB *Measure(s)*: TFTs per outpatient visit (mean, range)	0.17 (0.12–0.22) (I) 0.05 (0.04–0.06) (C)	0.13 (0.11–0.17) (I) 0.06 (0.05–0.08) (C)		The mean number of TFTs per outpatient visit decreased by 21.9% in the I group (p<0.01) and increase by 20.8% (not significant) in the C group. Similar results were obtained for the other tests for outpatients but not for inpatients (data NR).
Schectman *et al*,^33^ USA	1425 patients, 30 clinicians (group distribution not given)	*Intervention(s)*: R and R+FB *Measure(s)*: Compliance Mean (SE) number of TFTs ordered per patient	68% (R) 65% (R+FB) 1.68 (0.04)	81% (R) at 6 months 77% (R) at 12 months 64% (R+FB) at 6 months 80% (R+FB) at 12 months 1.37 (0.03) at 2/12 (following EM) 1.32 (0.05) at 6-month follow-up 1.49 (0.04) at 1-year follow-up		Significant increase in compliance in the reminder group (p=0.05) but not in the R+FB group; however, after excluding an outlier both groups had similar increase in compliance (77% vs 80%, p=0.39). The mean number of TFTs ordered per patient also decreased significantly (no p value given) but increased again at 1-year follow-up (only data combining both groups provided). TSH levels increased significantly while TT4 and T3RU decreased but no details are given.
Tomlin *et al*,^36^ New Zealand	GPs: 3140 (I) 2443 (C)	*Intervention(s)*: E+G+FB *Measure(s)*: Tests per year per GP: TSH: FT4: FT3: Total number of TFTs: Ratios of different TFTs: TSH/FT4 TSH/FT3 Expenditure (%)	223.6 (I) 33.8 (C) 144.2 (I) 29.1 (C) 41.6 (I) 11.0 (I) NR 2.4:1 7.1:1 NR	215.2 (I) 32.0 (C) 80.7 (I) 25.3 (C) 26.6 (I) 9.4 (C) 21% decrease (I) NR (C) 3.0:1 8.5:1 −19.8% (I) −9.5% (C)		TSH showed small decrease (4%, p<0.01) in the I group and no change in the C group (p<0.11). FT4 and FT3 decreased by 44.1% and 36.0%, respectively (p<0.01) in the I group and 13.1% and 14.6%, respectively, in the C group (p<0.01). In the I group, the proportion of TSH as the sole test ordered increased from 43.2% to 65.2% (p<0.001). Ratios of TSH to FT4 increased from 2.4:1 to 3.0:1 and TSH to FT3 from 7.7:1 to 8.5:1. Simultaneous testing of TSH and FT4 and/or FT3 decreased by 41.1% and there was a decrease in the net TFT expenditure (no p values given).
Wong *et al*,^42^ USA	NR	*Intervention(s)*: G+TRF *Measure(s)*: tests per month	*Intervention tests (months to intervention)*:* T4 (RIA) and T3RU: 9 months : 1100 6 months: 1150 3 months: 1100 TSH: 9 months: 900 6 months: 1300 3 months: 900 T3 (RIA): 9 months: 950 6 months: 1000 3 months: 900 *Control tests*:* CK: 9 months: 1000 6 months: 980 3 months: 980 LDH: 9 months: 650 6 months: 700 3 months: 750	*Intervention tests (months after intervention was introduced)*:* T4 (RIA) and T3RU: At 2 months: 1000 4 months: 1000 6 months:1100 8 months:950 TSH: 2 months: 500 4 months: 500 6 months:600 8 months:500 T3 (RIA): 2 months: 200 4 months: 300 6 months: 400 8 months: 300 Control tests*: CK: 2 months: 1100 4 months: 700 6 months:1000 LDH: 2 months: 700 4 months: 500 6 months:700		Distributing guidelines through a bulletin alone failed to produce effect but in combination with request form redesign it led to restructuring of test ordering patterns with decrease of ‘complete’ thyroid panel and increase of hyperthyroid and hypothyroid panels and thyroid function screen. No changes in T4 (RIA) and T3RU but the number of T3 (RIA) and TSH tests ordered per month fell on the average to 38% and 61%, respectively, of the mean monthly rates at which these tests had been ordered in the preceding 18 months. No changes were observed in the ordering of the control tests. Statistical significance of the above results is NR. Not all data presented here!
Interrupted time series						
Horn *et al*,^45^	Average monthly orders per 1000 patient visits (TSH): 174.1 (I) 140.3 (C)	*Intervention*: display of cost of tests being ordered *Measure(s)*: comparison of change-in-slope of the monthly ordering rates between intervention and control physicians for 12 months preintervention and 6 months postintervention	*Per cent change in monthly order rates (TSH, preintervention)*: 0.2% (I) 0.1% (C)	*Per cent change in monthly order rates (TSH, postntervention)*: 0.5% decrease (I) 0.4% increase (C)		The difference in the rate of change preintervention to postintervention was 0.7% decrease in the I group and 0.3% increase in the C group; none of these results was significant at p value <0.002 (2-sided Bonferroni-adjusted p value). Across all 27 evaluated tests, a statistically significant modest decrease in ordering rates of intervention physicians compared with control physicians was observed in 5 tests.
Van Walraven *et al*,^39^ Canada	NR	*Intervention(s)*: G+CFP; G+TRF *Measure(s)*: avoided tests, utilisation rate and cost		*1 July 1991*: TSH: 1 per 100 persons* TT4: 1.2 per 100 persons*	*G+CFP*: TT4: 4359 (95% CI −14 to 23 430) tests avoided T3RU: 3073 (−28 to 18 153) tests avoided *G+TRF*: TSH: 2200 (95% CI −1638 to 6039) tests avoided	G+CFP led to 96% decrease in the TT4 utilisation (p=0.02) and decrease in T3RU. Guidelines plus removing TSH ‘tick box’ from the request form resulted in 12% decrease in TSH utilisation (p=0.03). All interventions together resulted in a decrease of 626 098 tests, which saved $2 010 400, including $29 664 in the final year.
Before and after study designs						
Adlan *et al*,^22^ UK	Admissions: 1593 (pre) 1176 (post)	*Intervention(s)*: G *Measure(s)*: proportion of admitted patients offered TFTs	53.8% (857 out of 1593)	21.7% (255 out of 1176)		Significant reduction (32.1%, p<0.001) in the proportion of admitted patients offered TFTs
Chu *et al*,^24^ Australia	ED visits: 24 652 (pre) 25 576 (post)	*Intervention(s)*: G *Measure(s)*: mean number of TFTs ordered per 100 ED visits	2.2 (20-week preintervention period)	1.6 (20-week postintervention period)		Significant reduction in the mean number of TFTs (0.6 tests per 100 ED presentations, p=0.001) The mean number of all blood tests ordered per 100 ED presentations fell by 19% (p=0.001) and the mean cost fell by 17% (p=0.001).
Cipullo and Mostoufizadeh,^39^ USA	NA	*Intervention(s)*: G *Measure(s)*: mean tests utilisation (T3 per discharge)	0.006 (1 year before)	0.005 (1 year after)		The number of T3 uptake per discharge decreased by 17%. Most of the other targeted tests also showed decline in utilisation. Statistical significance NR.
Dowling *et al*,^26^ USA	Patient visits: 10 961 (pre), 6606 (post),3024 (at 5 months follow-up)	*Intervention(s)*: E+FB *Measure(s)*: proportion of indicated TSH (out of all TSHs performed) Rate of TSHs ordered per patient visit (total number of TSHs and visits) Rate of indicated TSH per visit Rate of non-indicated TSHs per visit	28% (25 of 90) 1 per 122 (90 in 10 961) 1 per 438 1 per 169	65% (15 of 23) 43% (9 of 21) (at 5 months follow-up) 1 per 287 (23 in 6 606) 1 per 178 (21 in 3 024) (at 5 months follow-up) 1 per 440 1 per 336 (at 5 months follow-up) 1 per 826 1 per 252 (at 5 months follow-up)		The proportion of indicated TSHs increased significantly (p<0.001) while TSHs per patient visit decreased significantly (p<0.0001) in the intervention period but both showed some decline at 5 months follow-up. The rate of indicated TSHs per visit did not change significantly while the rate of non-indicated TSHs per visit decreased drastically in the intervention period but increased again at follow-up. Data for the control test, CBC with differential, is not shown here but the rate of ordering showed steady decline even in the follow-up and the rates of ordering both indicated and non-indicated CBCs decreased at the end of the intervention, although the statistical significance of these results was NR.
Emerson and Emerson,^27^ USA	Unclear	*Intervention(s)*: TRF *Measure(s)*: number of tests/panels ordered preintervention to postintervention	TSH: 5300* FT4: 750 TT4: 1700 TT3: 800 FTI/T3RU: 900 TFT cascade: NA Combined TSH and cascade: 5250	TSH: 3000* FT4: 1450 TT4: 200 TT3: 500 FTI/T3RU: 100 TFT cascade: 1700 Combined TSH and cascade: 4750		TFT testing decreased significantly (p<0.01) with a shift towards FT4 and thyroid cascade. Across all tests, the total number of tests remained the same (due to an increase in the number of patients) but the number of tests per patient visit showed significant decrease (p<0.01).
Feldkamp and Carey,^28^ USA	Sequential TFT requests: 1000 (pre) 463 (post, 1 year) 625 (post, 3 years)	*Intervention(s)*: G *Measure(s)*: percentage of different TFTs and combinations:	*Prealgorithm*:	*1 year*	*2 years*	‘TSH only’ and DRTSH accounted for 92.4% of all TFTs 3 years after the introduction of the algorithm. The other combinations gradually decreased. However, the statistical significance of these results is NR.
		TFT only	33.3%	44.8%	32.2%	
		DRTSH algorithm	–	24.4%	60.2%	
		TSH+TT4+T3RU	16.6%	3.9%	1.3%	
		TT4 only	10.0%	4.8%	0.8%	
		TT4+T3RU	6.8%	2.8%	1.1%	
		Others (including TT3)	–	–	3.0%	
		TFTs per 1000 patients:	Prealgorithm:	Postalgorithm: DRTSH:	Tests/1000	
		TSH	832	982	1000	
		TT4	667	216	202	
		T3RU	234	159	202	
		Total	1 733	1359	1404	
		Difference	–	374	329	
Grivell *et al*,^29^ Australia	NR	*Intervention(s)*: FB *Measure(s)*: ratio of thyroxin requests postintervention to preintervention	NA	1.20		Thyroxin requests in the postintervention period were 1.2 times the requests in the preintervention period but the statistical significance of this result was NR.
Hardwick *et al*,^44^ Canada	NR	*Intervention(s)*: G+CFP *Measure(s)*: proportion (number) of different TFTs:	*1974/75*	*1976/77*	*1978/79*	Overall decline from baseline to 3 years postintervention (1978/79) with shift towards TT4 which accounted for 80.4% of all TFT investigations in the last period. Expenditure also decreased to 34% of the expected charges by the end of the study period. The statistical significance of these results was NR.
		TT3	21.8% (29 004)	19.0% (35 101)	4.7% (7502)	
		TT4	51.8% (68 912)	50.8% (93 988)	80.4% (128 343)	
		ETR	26.4% (35 183)	30.2% (55 798)	14.9% (23 703)	
		Total	100% (133 099)	100% (184 887)	100% (159 548)	
			*1975/76*	*1977/78*		
		TT3	20.4% (33 334)	11.8% (19 255)		
		TT4	49.6% (81 004)	62.3% (101 805)		
		ETR	29.8% (48 832)	25.9% (42 346)		
		Total	100% (163 170)	100% (163 406)		
Horn *et al*,^45^	Physicians: 153 (I) 62 (C)	*Intervention(s)*: Display of cost of tests being ordered (computer-based ordering system) *Measure(s)*: difference in per cent change in monthly orders between I and C group (orders per 1000 patient visits)	*Baseline average monthly order rate (orders per 1000 visits)*: 174.1 (I) 140.3 (C) *Per cent change in monthly order rate*: 0.2% (I) 0.1% (C)	*Per cent change in monthly order rate*: −0.5% (I) 0.4% (C)	*Difference*: −0.7% (I) 0.3% (C)	Monthly order rates for TSH decreased slightly in the I group and increased in the C group but the difference was not statistically significant (p=0.04; because 27 tests were analysed, the study used a 2-sided Bonferroni-adjusted p value of <0.002 to determine statistical significance).
Larsson *et al*,^30^ Sweden	19 primary care centres	*Intervention(s)*: E *Measure(s)*: mean ratios of the requests for related tests:	*1996*	*1997*		Significant decrease in TT3/TSH (difference between mean ratios 0.073, SD=0.089, p=0.0012) and non-significant decrease in FT4/TSH (difference 0.032, SD=0.116, p=0.13). As recommended, the ratio of TSH to all TFTs increase significantly (difference −0.017, SD=0.041, p=0.048).
		TT3/TSH	0.129	0.056		
		FT4/TSH	0.333	0.301		
		TSH/total number of TFTs	0.124	0.141		
Mindemarkand Larsson^31^ (follow-up of Larsson 1999)		Median ratios:	*1997*	*2004*		7 years after the intervention TT3/TSH and (TT4+FT4)/TSH were not significantly different from those at the end of the original study period, thus showing no decay in the intervention's effect. However, THS/all TFTs showed slight decrease (difference −0.043) which was statistically significant (p<0.05). Most of the other tests’ ratios also remained stable.
		TT3/TSH	0.029	0.022		
		(TT4+FT4)/TSH	0.273	0.237		
		TSH/all TFTs	0.115	0.072		
Nightingale *et al*,^32^ UK	Number of patients: 654 (before) 833 (after)	*Intervention(s)*: PMS+E+FB *Measure(s)*: change in compliance	55%*	85%*		Compliance of TSH requests increased but results are given only as a graph and the statistical significance is NR. Across all tests, the total number of tests requested per patient day declined by 17% (p<0.001).
Rhyne and Gehlbach,^43^ Canada	NR	*Intervention(s)*: G+E *Measure(s)*: TFPs per 100 encounters Proportion of: ‘high indications’ ‘low indications’	*October to December 1976* 1.00 *January to March 1977* Approximately 1.10 45% 29%	*June to August 1977* Approximately 0.70 *September to November 1977* Approximately 1.00 53% 19%		Significant decrease in the number of TFP ordered per encounter in the 3 months after the intervention (p<0.05) but return to baseline in the following 3 months. Results given only as a graph. The proportion of FTP for ‘high indications’ increased and that for ‘low indications’ decreased but was not statistically significant (p<0.05). Senior residents decreased their TFP ordering rate while that of junior residents increased (p<0.05).
Schectman *et al*,^33^ USA	1425 patients, 30 clinicians (group distribution not given)	*Intervention(s)*: EM *Measure(s)*: compliance rate Mean (SE) number of TFTs ordered per patient	35% 1.68 (0.04)	67% at 2 months (following EM) 1.37 (0.03) at 2 months (following EM) 1.49 (0.04) at 1 year follow-up		Significant increase in compliance after EM (p<0.0001) The mean number of TFTs ordered per patient decreased significantly following EM (p<0.0001) and showed further decline at 6 months after the subsequent interventions (*see under Non-randomised controlled studies above*) but increased again at 1 year follow-up. The educational intervention had greater impact on nurses and physician assistants than physicians (absolute increase in compliance 63% vs 28%).
Stuart *et al*,^34^ Australia	NR; annual census of 42 500 patients	*Intervention(s):* E+G+FB *Measure(s)*: mean cost of TFT (in $A) per patient	0.426	0.047		TFT ordering decreased by 89% and showed a significant decrease in cost per patient (−89% difference (95% CI −55% to −123%; p<0.001). Across all tests, there was 40% decrease in the ordering of tests with test utilisation falling from a mean of $39.32/patient to $23.72/patient (p<0.001). Tests not allowed to be ordered for ED patients, such as TFT, showed the greatest decrease. The effect was sustained at 18 months follow-up.
Toubert *et al*,^37^ France	800-bed hospital with annual census of 25 266 inpatients and 242 013 outpatients	*Intervention(s)*: G+R Measure(s): number of TFTs:	*1996*	*1997*	*1998*	A substantial decrease in the total number of TFTs mainly due to a decrease in the number of FT3 and FT4; a decrease in the relative proportion of FT3. Single TSH order forms increased from 23% to 50%, while TSH+FT4+FT3 decreased. The statistical significance of these results was NR. The percentage of appropriate tests increased from 42.5% (95%CI 39.9% to 45.1%) in 1996 to 72.4% (95%CI 69.8% to 75%) in 1997 (p<0.0001) but there was some decrease in 1998 (no p value given). (Data for thyroid antibodies is not presented here.)
		Total TSH tests	1329	1119	1062	
		Total FT4 tests	1011	535	539	
		Total FT3 tests	715	247	226	
		Total number of TFTs	3145	1901	1827	
		Patterns:				
		Single TSH	305	563	512	
		TSH+FT4	319	313	333	
		TSH+FT3	23	25	20	
		TSH+FT4+FT3	682	218	197	
		FT4+FT3	10	4	9	
		All TFT request forms	1339	1123	1071	
		Appropriateness:	42.5%	72.4%	70.7%	
van Gend *et al,*^38^ The Netherlands	NR	*Intervention(s)*: TRF+FB *Measure(s)*: ratio of FT4 (removed from) to TSH (left on the request form)	*1992* 0.96 (2498:2608)	*1993* 0.31 (1180:3747) *1994* 0.28 (1436:5040)		There was a decrease in the FT4:TSH ratio indicating that the intervention had impact on test ordering patterns but the statistical significance of the results was NR.
Vidal-Trécan *et al*,^40^ France	*June 1995*: all TFTs: 27 945	*Intervention(s)*: E+G+TRF *Measure(s)*: number (%) of TFTs	*June 1995*	*June 1998*		11% decrease in the total number of TFTs even though the number of admissions increased by 2% and the number of outpatient visits by 6%. The proportion of FT4 tests remained the same (33%); the proportion of FT3 measurements decreased by 6% and the proportion of TSH tests increased. The statistical significance of the results was NR.
		Total TFTs	100% (27 945)	88.7% (24 794)		
		FT3	20% (5491)	14% (3534 of 24 794)		
		TT3	1% (339)	1% (371 of 24 794)		
		FT4	33% (9301)	33% (8125 of 24 794)		
		TT4	2% (478)	1% (238 of 24 794)		
		TSH	44% (12 336)	51% (12 526 of 24 794)		
Willis and Datta,^41^ UK	An average of 950 patients and 309 thyroid profiles per month	*Intervention(s)*: E+G *Measure(s)*: mean (SD) number of TFT profiles per admission	0.32 (0.05)	0.08 (0.01)		Significant decrease in the number of requested thyroid profiles (p<0.001). Across all tests, a significant change between the total number of sets requested per admission before (7.5 (0.87)) and after the intervention (5.9 (0.33)), p<0.001.

*Approximate reading off a graph.

C, control group; CBC, complete blood count; CFP, changes to funding policy; CK, creatinine kinase; CV, coefficient of variation; DRTSH, directed thyroid testing algorithm; E, education; ED,emergency department; EM, educational memorandum; ETR, effective thyroxin ratio; FB, feedback; FBC, feedback on cost; FBY, feedback on yield; FT3, free tri-iodothyronine; FT4, free thyroxine; FTI, free thyroid index; G, guidelines/protocol; GCR, guideline conformity rate, the proportion of TFTs ordered in accordance with the guidelines; GP, general practitioner; I, Intervention group; LDH, lactic dehydrogenase isoenzyme; NA, not available; NR, not reported; PMC, pocket memory card; PMS, protocol management system; R, reminders; T3 (RIA), tri-iodothyronine radioimmunoassay; T3RU, tri-iodothyronine resin uptake; T4 (RIA), thyroxin radioimmunoassay; TFP, thyroid function panel; TFTs, thyroid function tests; TRF, test request form redesign; TSE, test-specific education; TSH, thyroid-stimulating hormone; TT3, total tri-iodothyronine; TT4, total thyroxin.

### Study quality

The results from the methodological quality assessment are presented in table 5. In terms of study design, four were RCTs,^10^ ^23^ ^25^ ^35^ five studies were non-randomised controlled studies,^17^ ^18^ ^33^ ^36^ ^42^ two were interrupted time series^39^ ^45^ and the remaining were single-group studies with just one time point measurement before and after. Most of the studies were of poor or moderate quality; the main issues being selection bias, lack of blinding and failure to control for confounders.

**Table 5:** Results from the methodological quality assessment of the included studies using the EPHPP tool

Study	Selection bias	Study design	Confounders	Blinding	Data collection method	Withdrawals and dropouts	Global rating
Randomised controlled trials							
Baker *et al*^23^	Strong	Strong	Strong	Moderate	Strong	Strong	Strong
Daucourt *et al*^25^	Moderate	Strong	Strong	Strong	Moderate	Strong	Strong
Thomas *et al*^10^	Strong	Strong	Strong	Strong	Strong	Strong	Strong
Tierney *et al*^35^	Strong	Strong	Strong	Moderate	Strong	Strong	Strong
Non-randomised controlled studies							
Berwick and Coltin^17^	Moderate	Strong	Moderate	Weak	Strong	Weak	Weak
Gama *et al*^18^	Weak	Moderate	Weak	Weak	Strong	Strong	Weak
Schectman *et al*^33^	Moderate	Strong	Weak	Moderate	Strong	Strong	Moderate
Tomlin *et al*^36^	Strong	Strong	Weak	Moderate	Strong	Moderate	Moderate
Wong *et al*^42^	Weak	Moderate	Weak	Moderate	Strong	Weak	Weak
Interrupted time series							
Horn *et al*^45^	Moderate	Moderate	Moderate	Weak	Strong	Moderate	Moderate
van Walraven *et al*^39^	Strong	Moderate	Moderate	Strong	Strong	Strong	Strong
Before and after studies							
Adlan *et al*^22^	Weak	Moderate	Weak	Moderate	Strong	Weak	Weak
Chu *et al*^24^	Weak	Moderate	Weak	Moderate	Strong	Weak	Weak
Cipullo and Mostoufizadeh^19^	Weak	Moderate	Weak	Weak	Weak	Weak	Weak
Dowling *et al*^26^	Weak	Moderate	Weak	Weak	Weak	Strong	Weak
Emerson and Emerson^27^	Weak	Moderate	Weak	Weak	Strong	Weak	Weak
Feldkamp and Carey^28^	Weak	Moderate	Weak	Weak	Strong	Weak	Weak
Grivell *et al*^29^	Weak	Moderate	Weak	Weak	Strong	Weak	Weak
Hardwick *et al*^44^	Strong	Moderate	Moderate	Strong	Strong	Strong	Moderate
Larsson *et al*^30^	Moderate	Moderate	Weak	Moderate	Strong	Strong	Moderate
Nightingale *et al*^32^	Weak	Moderate	Weak	Weak	Strong	Weak	Weak
Rhyne and Gehlbach^43^	Weak	Moderate	Weak	Moderate	Strong	Weak	Weak
Stuart *et al*^34^	Weak	Moderate	Weak	Moderate	Moderate	Moderate	Weak
Toubert *et al*^36^	Weak	Moderate	Weak	Moderate	Moderate	Weak	Weak
van Gend *et al*^38^	Strong	Moderate	Weak	Moderate	Strong	Strong	Moderate
Vidal-Trécan *et al*^40^	Moderate	Moderate	Moderate	Moderate	Strong	Strong	Strong
Willis and Datta^41^	Weak	Moderate	Weak	Moderate	Strong	Weak	Weak

### Effectiveness of the interventions

#### Single-mechanism interventions

Fourteen studies^10^ ^17–19^ ^22^ ^24^ ^25^ ^27–31^ ^33^ ^35^ ^45^ evaluated the effectiveness of the following single-mechanism interventions (tables 2–4): educational programmes (one controlled and two before and after studies),^17^ ^30^ ^33^ guidelines and protocols (four before and after studies),^19^ ^22^ ^24^ ^28^ reminders (two RCT and one controlled study),^10^ ^25^ ^33^ decision-making tools (two RCTs, one interrupted time series and one before and after study),^25^ ^27^ ^35^ ^45^ and audit and feedback (two controlled and two before and after studies).^17^ ^18^ ^29^ ^33^ The study by Schectman *et al*^33^ was a two-stage study before and after design in the first part and a comparative design in the second. The majority of the evaluated single-mechanism interventions were effective in decreasing test-related expenditure,^35^ the volume of test ordering,^17^ ^18^ ^22^ ^24^ ^27–29^ ^33^ changing the pattern of TFT ordering^19^ ^27^ ^28^ ^30^ ^31^ or increasing compliance^25^ ^33^ in accordance with the recommended practice.

Two of these studies reported data on test ordering once the intervention was discontinued. Mindemark and Larsson^31^ investigated the effect of the 2-day educational programme originally evaluated by Larsson *et al*^30^ in a before and after study. Eight years after the programme was delivered, they found that the ratios between pairs of different TFTs was similar to that measured at the end of the original study (1 year after the delivery of the programme). Only the ratio ‘TSH:all TFTs’ showed slight but statistically significant decrease which the authors explained with the recommendation given to participants to analyse TSH in elderly patients who had not been tested in the previous 2–3 years (table 4). Although impressive, the observed result is difficult to explain with the educational programme alone as other contextual factors are likely to have contributed to the persistence of the effect.

Tierney *et al*^35^ reported that 6 months after the intervention (display of computer-generated probability estimates evaluated in an RCT) the difference between intervention and control group has disappeared and the main outcome—charges per scheduled visit—has returned to baseline (table 4).

Three studies reported unsuccessful interventions: an RCT of good methodological quality demonstrated that a reminder in the form of a memorandum pocket card was unsuccessful in increasing compliance with the recommended thyroid testing strategy;^25^ a poor quality before and after study showed that monthly feedback given to consultants for a period of 1 year was unable to decrease the ordering rates of a number of laboratory tests;^29^ and an interrupted time series of moderate methodological quality demonstrated that displaying the cost of tests at the time of ordering was moderately effective in a small number of tests but did not affect the ordering of TFTs.^45^ The former two studies were conducted in a hospital setting and the latter was a primary care study.

The authors of the before and after study explained the failure of the intervention by the prevailing institutional culture; the fact that the clinical units ordering the largest proportion of tests showed little concern and attributed their requesting pattern to clinical workload and the nature of their patients; and by the fact that the feedback was provided to consultants only, on their request, while many of the tests were ordered by junior doctors unaffected by the intervention.^29^ The authors of the interrupted time series study surveyed all intervention and non-intervention physicians to investigate their perceptions regarding the intervention and healthcare costs in general. They found that while nearly all participants endorsed the need for cost containment and found the display of costs informative, 50% of them reported that the displays ‘rarely’ or ‘never’ impacted their decision to order the tests.^45^

Berwick and colleagues who compared two different types of feedback, on cost and on yield, with test-specific education reported mixed results. Across all tests, only feedback on cost showed statistically significant effect, whereas for the TFTs test-specific education had the largest effect. With regard to reducing variability in test ordering, the two forms of feedback but not education led to a positive change. The statistical significance of the results specific to TFTs, however, was not reported.^17^

#### Multifaceted interventions

Sixteen studies evaluated interventions that relied on more than one mechanism to change test ordering behaviour (tables 2–4).^10^ ^23^ ^25^ ^26^ ^32–34^ ^36–44^ Ten of them combined the introduction of guidelines or protocols with audit and feedback,^23^ education,^41^ ^43^ redesign of a test request form,^39^ ^42^ changes to funding policy,^39^ ^44^ education plus audit and feedback,^34^ ^36^ reminders,^37^ or education plus a test request form.^40^ Of the remaining six studies, three evaluated the combination of audit and feedback with education,^26^ reminders^10^ or a problem-oriented test request form;^38^ one study evaluated the effectiveness of a computer-based protocol management system enhanced with audit and feedback and education;^32^ one compared an educational memorandum followed by a reminder with the same educational memorandum followed by a reminder and feedback;^33^ and one compared a combination of pocket memory card (reminder) and redesign of test request form with the same single-mechanism interventions and usual practice defined as simple diffusion of guidelines.^25^

With the exception of one study,^23^ all reported that the evaluated multifaceted interventions were effective in decreasing the volume of test ordering,^10^ ^23^ ^26^ ^33^ ^36^ ^37^ ^39–41^ ^43^ ^44^ changing the pattern of test ordering in accordance with the recommended practice,^26^ ^33^ ^37–40^ ^42–44^ avoiding unnecessary testing^39^ and/or decreasing test-related expenditure.^34^ ^36^ ^39^

Two before and after studies reported data on test ordering once the intervention was discontinued.^26^ ^33^ Dowling and colleagues evaluated the effectiveness of education plus feedback and measured the rate of TSH ordered per patient visit and the indicated and non-indicated TSH per visit. Despite the initial statistically significant effect, 5 months after the intervention was discontinued, all indicators showed some decline. Schectman *et al*^33^ evaluated the effect of educational memorandum followed by reminder or reminder and feedback on compliance and the mean number of TFTs ordered per patients. The interventions increased compliance and led to significant decrease in test ordering which continued 6 months after the interventions but increased again at 1 year follow-up (table 4).

The study that reported an unsuccessful intervention was an RCT of good methodological quality and was conducted in primary care. It targeted the use of five frequently ordered laboratory tests including TSH and FT4 and evaluated the effectiveness of a combination of guidelines and feedback. The authors explained the failure of the intervention by the following: the feedback was provided for 1 year only, the participating practices did not volunteer to take part in the study and the guidelines might not have been sufficient to predispose physicians to change their test ordering behaviour.^23^

Owing to the significant heterogeneity and poor methodological quality of the studies, we deemed pooling the results inappropriate and were unable to use statistical methods to investigate the impact of various study and intervention characteristics on the reported outcomes. Visual inspection of the data suggests, however, that differences such as intervention type, study design, setting and year of publication have little or no impact on the reported effectiveness. We created a spreadsheet which can be used by the readers to explore this themselves by sorting the results according to different study characteristics (see online supplementary appendix 2).

10.1136/bmjopen-2015-010065.supp2 Supplementary appendix 2

## Discussion

### Main findings

This systematic review of behaviour change interventions designed to modify the ordering of TFTs found 27 studies. Several intervention types were evaluated including education, guidelines and protocols, audit and feedback, decision-making tools, changes to funding policy and reminders, either alone or in combination, in either primary or hospital care, and targeting clinicians of different seniority. Most of the studies were of poor or moderate quality and many of the interventions were poorly described.

In these studies, it appears that behaviour change interventions were, in general, effective in reducing the volume, changing the pattern of test ordering, improving compliance with guidelines or reducing the cost of TFTs ordered. Whether such changes reflect more appropriate test ordering, however, was unclear as in the majority of studies measures of appropriateness were undefined, and thus unreported. No study investigated directly any impact on patient outcomes. Only five studies observed the effect of the intervention on test ordering for more than 12 months^28^ ^34^ ^36^ ^37^ ^44^ and only four reported the persistence of effect once the intervention had ended,^26^ ^30^ ^31^ ^33^ ^35^ of which three reported return to baseline or decline within 1 year.^26^ ^33^ ^35^

Although not the subject of an a priori subgroup analysis, multifaceted interventions did not appear to be more effective than single-mechanism ones. The specific type of intervention(s) appeared less important than the interaction between various intervention-specific variables and the implementation of the intervention in a specific context. For instance, feedback could be very successful if there was a strong institutional support for change^26^ or completely ineffective if the changes it was trying to introduce clashed with the dominant institutional culture.^29^ Even within a single study, the same intervention performed differently in different clinical circumstances (eg, inpatients vs outpatients)^18^ or different interventions seemed to be effective for different type of tests.^17^ ^45^ As Mindemark and Larsson^31^ put it “…the most decisive factor for the success of a strategy in optimizing test ordering is not the nature of the intervention itself, but rather its design and implementation in a given setting.” (p. 485)

The effectiveness of the interventions depended to some extent on the outcomes the researchers had chosen to measure. These outcomes reflected specific assumptions about what constitutes inappropriate test ordering and how this could be changed. ‘Appropriateness’ is to a large extent, a value judgement, incorporating elements of importance of the diagnosis, benefits from early diagnosis (and the converse, harms from delays in diagnosis), burden and unpleasantness of the test, plus economic considerations. These aspects were rarely—if ever—explicitly reported as part of the rationale for the intervention and selection of the outcome measure. It was simpler to examine the volume of testing or the shift in the pattern of TFTs ordered which most studies did, but clinically, this is a blunt measure of appropriateness.

For instance, in one study a one-off educational event encouraged GPs to use TSH as a single first-line test^30^ and the intervention was reported to have a long-term effect.^31^ The following ratios were used as an outcome measures: ‘TSH:all TFTs’ (which was expected to increase) and ‘T3:TSH’ and ‘FT4:TSH’ (which were expected to decrease as a result). Therefore, the intervention did not address inappropriate ordering of TSH and the chosen outcomes could not capture a possible ‘shift’ where doctors ordered inappropriately more TSHs while ordering fewer T3 and FT4 tests. An interrupted time series analysis which investigated the effect of a series of interventions over a period of several years clearly demonstrates such a possibility.^39^

### Strengths and limitations of study

We conducted the current systematic review using the methods recommended by the Cochrane Collaboration working to a prespecified protocol and consider our findings to be robust. This is the first review focusing specifically on the effectiveness of interventions designed to reduce unnecessary ordering of TFTs. The identified evidence is directly relevant to this particular test ordering behaviour and could be used to guide the design and implementation of future intervention programmes as well as the development of research projects that could address the identified gaps in knowledge. The main limitation is that the quality of evidence did not allow strong conclusions and more specific recommendations to be made. Furthermore, the disparate methods, populations of study, interventions and outcome measures made pooled synthesis of results impossible. Thus, we have chosen to present the results as a narrative synthesis. Similarly, although we strongly suspect that publication bias and selective reporting of outcomes may be operating, particularly for the non-randomised study designs, we could neither investigate nor attempt to quantify the potential impact. We think it is likely that publication bias has exaggerated the results, but is highly unlikely to completely account for the overall beneficial pattern observed. That the more rigorous designs gave less marked and even negative results (tables 3 and 4 and online supplementary appendix 2) adds a note of caution however.

### Comparison with other studies

We are not aware of another systematic review with a similar focus. Other systematic reviews have examined the effectiveness of behavioural interventions designed to influence test ordering as a whole.^12–16^ Our review focused on a specific diagnostic scenario—the use of laboratory tests to diagnose and monitor thyroid dysfunction.

### Clinical interpretation of the results

Superficially, based on the predominant pattern of favourable results from the included studies, there would seem to be little doubt that where there is evidence that TFTs ordering needs to be modified, the interventions employed in the included studies could be used to effectively reduce volume, improve compliance, change the pattern of testing or reduce cost. However, we believe some caution is required. The most fundamental issue is that in many included studies, the detail about the nature of the intervention is insufficient for implementation; the situation is particularly acute where the target behaviour is appropriateness, because the value of achieving compliance is completely dependent on the definition of appropriateness being used and how it was derived. The problem concerning insufficient definition of the intervention has been noted before and is, we believe, particularly relevant here.^48^ Although, additional details may exist outside the published paper and be obtained through personal contact with the investigators, much information about the interventions is likely to be unavailable, particularly for older studies.

Similarly, the current applicability of many of the interventions can be questioned. The circumstances operating in many of the studies may not be similar to the challenges today. A simple example from the study by Thomas *et al*,^10^ clearly applicable to UK primary care, is that the rates of test ordering were in the region of 800 per 10 000 practice patients, whereas the equivalent rates in a recent study in the South West were 2500 per 10000.^5^ The importance of this is accentuated by the fact that interventions do not seem to have been designed in the light of investigations to understand the origin of the difficulties underlying the behaviour they were attempting to address. Thus, in primary care, it is widely accepted that the reason why GPs order tests is frequently not for medical reasons,^49^ yet most interventions we encountered, such as education and guidelines, assume that lack of medical knowledge is the underlying difficulty. Our own investigations of GPs' reasons why TFT ordering rates might vary, included many factors such as quality of computer systems, communication with hospital systems, general attitude to risk, involvement of other members of the primary care team in test ordering and patient expectations, all issues which are unlikely to have been addressed by any of the interventions we observed.^7^ Our concerns about openness to bias of the included studies and possibility of publication and outcome reporting bias reinforce our circumspection about whether the evidence reviewed is good enough to implement.

Given the fact that the majority of TFTs are ordered by GPs in the UK^5^ and that guidelines for the use of these tests in primary care already exist (though appear to be ineffective), interventions that raise clinicians' awareness of these guidelines, ‘translate’ them into easy to follow rules and embed them in decision aids are potentially effective combination. This review suggests that most interventions succeed (albeit in the limited way described above), so it is probable that an intervention can be designed that would work in UK primary care. Other factors will still be relevant, as highlighted by the qualitative study, such as lack of communication, problems with storage and retrieval of previous results, and lack of local protocols that structure the ordering of TFTs in accordance with the existing guidelines.^7^

### Conclusions and policy implications

The systematic review we have conducted indicates that behaviour change interventions can modify TFT ordering. While a starting point for implementation, we do not believe the evidence base is complete and strongly recommend further research. As well as overcoming the limitations highlighted concerning bias, improving details of the interventions to be implemented and improving applicability to current challenges, new research can also address questions barely touched on by the existing evidence base. Such questions include the effectiveness of interventions like computerised test ordering systems (order.com's) in primary care, how to maintain the effect on test ordering over several years and cost-effectiveness. The scale of the problem is important in this regard. While a few of the included studies had large effects, most only had small effects which would not be large enough to impact for instance on the sixfold variation in test ordering in primary care observed in the South West^5^ or the even more extreme variation observed across the UK.^6^

The current study also demonstrates that even though reviewing the evidence with the target behaviour clearly in mind is a more productive approach than looking at similar behaviour change interventions applied to a wide variety of targets, such an approach has limitations. For instance, many studies targeting a wide range of test ordering behaviour reported only average effect, even when it was clear that the effect on the ordering of different tests was quite different. This limited the number of studies available for inclusion and probably accounted for the small number of studies evaluating specific interventions such as those based on computerised test ordering systems. Moreover, even when the studies reported test-specific effects, they rarely investigated the reasons for this variation and failed to provide explanation of the observed differences. Given the poor methodological quality of many of the included studies, this made it difficult to draw reliable conclusions and make recommendations. This suggests that although similar reviews to this looking at the effectiveness of behaviour change interventions on modifying the ordering of other routine tests would be helpful, a novel approach may be necessary. Such approach could focus on similar test ordering behaviours rather than similar tests and could incorporate wider range of evidence able to demonstrate not only the effectiveness of different interventions but also to provide insight in the mechanisms behind specific behaviour modifications.
