# Supplementary material for: Effectiveness of interventions to reduce ordering of thyroid function tests: a systematic review
Source: BMJ Open. 2016 Jun 3;6(6):e010065. doi: 10.1136/bmjopen-2015-010065 (PMC4893867; doi:10.1136/bmjopen-2015-010065)
Supplement: Supplementary appendix 1 [file bmjopen-2015-010065supp_appendix1.pdf]

Appendix 1 Search strategies

MEDLINE

Database: Ovid MEDLINE(R) In-Process & Other Non-Indexed Citations and Ovid MEDLINE(R) <1946 to Present>

Search Strategy:

----

1 Thyroid Function Tests/ (12058)

2 Laboratories, Hospital/ut [Utilization] (191)

3 Clinical Laboratory Techniques/ut, sn (919)

4 Clinical Chemistry Tests/ec, ut [Economics, Utilization] (66)

5 Diagnostic Tests, Routine/ec, sn, td, ut [Economics, Statistics & Numerical Data, Trends, Utilization] (1995)

6 (thyroid function adj (test or tests or testing)).ti,ab. (2549)

7 (test* adj5 thyroid).ti,ab. (6241)

8 (TSH adj5 test*).ti,ab. (1321)

9 (T3 adj5 test*).ti,ab. (942)

10 (FT3 adj5 test*).ti,ab. (79)

11 (TT3 adj5 test*).ti,ab. (15)

12 (T4 adj5 test*).ti,ab. (810)

13 (FT4 adj5 test*).ti,ab. (161)

14 (TT4 adj5 test*).ti,ab. (36)

15 thyroid stimulating hormone test*.ti,ab. (21)

16 (triiodothyronine adj5 test*).ti,ab. (420)

17 (thyrotropin adj5 test*).ti,ab. (675)

18 TSH assay*.ti,ab. (330)

19 thyrotropin assay*.ti,ab. (82)

20 or/1-19 (21931)

21 (test* adj5 request*).ti,ab. (1987)

22 (test* adj2 practic*).ti,ab. (2997)

23 (test* adj5 order*).ti,ab. (18586)

24 (unnecessary adj5 test*).ti,ab. (1535)

25 (inappropriate* adj5 test*).ti,ab. (829)

26 (test* adj5 strateg*).ti,ab. (9713)

27 (test* adj5 guidelines).ti,ab. (3552)

28 (appropriate* adj5 test*).ti,ab. (10630)

29 (misuse adj4 test*).ti,ab. (164)

30 or/21-29 (47808)

31 Unnecessary Procedures/ec, ut, st [Economics, Utilization, Standards] (569)

32 30 and 31 (66)

33 30 and 20 (868)

34 32 or 33 (906)

****

Embase

Database: Embase <1974 to 2013 November 25>

Search Strategy:

----

1 Thyroid Function Tests/ (12224)

2 (thyroid function adj (test or tests or testing)).ti,ab. (3606)

3 (thyroid adj2 screening).ti,ab. (858)

4 (test* adj5 thyroid).ti,ab. (8381)

5 (TSH adj5 test*).ti,ab. (1921)

6 (T3 adj5 test*).ti,ab. (1337)

7 (FT3 adj5 test*).ti,ab. (145)

8 (TT3 adj5 test*).ti,ab. (26)

9 (T4 adj5 test*).ti,ab. (1134)

10 (FT4 adj5 test*).ti,ab. (278)

11 (TT4 adj5 test*).ti,ab. (37)

12 thyroid stimulating hormone test*.ti,ab. (30)

13 (triiodothyronine adj5 test*).ti,ab. (456)

14 (thyrotropin adj5 test*).ti,ab. (747)

15 TSH assay*.ti,ab. (451)

16 thyrotropin assay*.ti,ab. (98)

17 or/1-16 (21791)

18 (test* adj5 request*).ti,ab. (2721)

19 (test* adj2 practic*).ti,ab. (3808)

20 (test* adj5 order*).ti,ab. (23260)

21 (unnecessary adj5 test*).ti,ab. (2095)

22 (inappropriate* adj5 test*).ti,ab. (1129)

23 (test* adj5 strateg*).ti,ab. (11504)

24 (test* adj5 guidelines).ti,ab. (4942)

25 (appropriate* adj5 test*).ti,ab. (14872)

26 (misuse adj4 test*).ti,ab. (183)

27 or/18-26 (61598)

28 17 and 27 (366)

****

Cochrane library

Database: Cochrane Library

Date Run: 27/11/13 11:20:54.437

Description:

ID	Search	Hits
#1	MeSH descriptor: [Thyroid Function Tests] explode all trees	535

#2 MeSH descriptor: [Clinical Chemistry Tests] explode all trees 6165

#3 MeSH descriptor: [Diagnostic Tests, Routine] explode all trees 291

#4 ("thyroid function" next (test*)):ti,ab 76

#5 (test* near/5 thyroid):ti,ab 161

#6 (TSH near/5 test*):ti,ab 53

#7 (T3 near/5 test*):ti,ab 59

#8 (FT3 near/5 test*):ti,ab 2

#9 (TT3 near/5 test*):ti,ab 1

#10 (T4 near/5 test*):ti,ab 38

#11 (FT4 near/5 test*):ti,ab 4

#12 (TT4 near/5 test*):ti,ab 2

#13 "thyroid stimulating hormone test*":ti,ab 1

#14 (triiodothyronine near/5 test*):ti,ab 10

#15 (thyrotropin near/5 test*):ti,ab 18

#16 "tsh assay*":ti,ab 8

#17 "thyrotropin assay*":ti,ab 0

#18 #1 or #2 or #3 or #4 or #5 or #6 or #7 or #8 or #9 or #10 or #11 or #12 or #13 or #14 or #15 or #16 or #17 7179

#19 ("test* near/5 request*"):ti,ab 0

#20 ("test* near/5 practic*"):ti,ab 0

#21 ("test* near/5 order*"):ti,ab 0

#22 (unnecessary near/5 test*):ti,ab 45

#23 (inappropriate* near/5 test*):ti,ab 33

#24 (test* near/5 strateg*):ti,ab 667

#25 (test* near/5 guidelines):ti,ab 138

#26 (appropriate* near/5 test*):ti,ab 564

#27 (misuse near/4 test*):ti,ab 13

#28 #19 or #20 or #21 or #22 or #23 or #24 or #25 or #26 or #27 1408

#29	#18 and #28	35
