# Supplementary material for: Effectiveness of interventions to reduce ordering of thyroid function tests: a systematic review
Source: BMJ Open. 2016 Jun 3;6(6):e010065. doi: 10.1136/bmjopen-2015-010065 (PMC4893867; doi:10.1136/bmjopen-2015-010065)
Supplement: Supplementary appendix 2 [file bmjopen-2015-010065supp_appendix2.pdf]

Instructions: Place the cursor on the first row cell to see explanations. Highlight area to be sorted including column headings (B2:S28). Click on Sort function in Data menu. Choose desired heading and observe pattern of results when sorted by study feature.

Test numbers or rates

Study	Year	Country	Setting	Intervention	Reminders	Education	Feedback	Decision tools	Funding	Guidelines	Single	Just TFTs	Design	Outcome
Adlan 2011	2011	UK	Hosp	Guidelines	N	N	N	N	N	Y	S	Y	BA	Numbers/rate
Baker 2003	2003	UK	PC	Education and guidelines	N	Y	N	N	N	Y	M	N	RCT	Numbers/rate
Berwick 1986	1986	USA	PC	Feedback on cost	N	N	Y	N	N	N	S	N	CS	Numbers/rate
Berwick 1986	1986	USA	PC	Feedback on yield	N	N	Y	N	N	N	S	N	CS	Numbers/rate
Berwick 1986	1986	USA	PC	Education (test specific)	N	Y	N	N	N	N	S	N	CS	Numbers/rate
Berwick 1986	1986	USA	PC	Feedback on cost	N	N	Y	N	N	N	S	N	CS	CV
Berwick 1986	1986	USA	PC	Feedback on yield	N	N	Y	N	N	N	S	N	CS	CV
Berwick 1986	1986	USA	PC	Education (test specific)	N	Y	N	N	N	N	S	N	CS	CV
Chu 2013	2013	Australia	Hosp	Guidelines	N	N	N	N	N	Y	S	N	BA	Numbers/rate
Cipullo 1996	1996	USA	Hosp	Guidelines	N	N	N	N	N	Y	S	N	BA	Numbers/rate
Daucourt 2003	2003	France	Hosp	Pocket memory card	Y	N	N	N	N	N	S	Y	RCT	Appropriateness/compliance
Daucourt 2003	2003	France	Hosp	Request form redesign	N	N	N	Y	N	N	S	Y	RCT	Appropriateness/compliance
Daucourt 2003	2003	France	Hosp	Memory pocket card and request form redesign	Y	N	N	Y	N	N	M	Y	RCT	Appropriateness/compliance
Dowling 1989	1989	USA	PC	Education and feedback	N	Y	Y	N	N	N	M	N	BA	Numbers/rate
Dowling 1989	1989	USA	PC	Education and feedback	N	Y	Y	N	N	N	M	N	BA	Appropriateness/compliance
Emerson 2001	2001	USA	PC	Request form redesign	N	N	N	Y	N	N	S	N	BA	Numbers/rate
Emerson 2001	2001	USA	PC	Request form redesign	N	N	N	Y	N	N	S	N	BA	Pattern
Feldkamp 1996	1996	USA	Hosp	Guidelines	N	N	N	N	N	Y	S	Y	BA	Numbers/rate
Feldkamp 1996	1996	USA	Hosp	Guidelines	N	N	N	N	N	Y	S	Y	BA	Pattern
Gama 1991	1991	UK	Hosp	Feedback	N	N	Y	N	N	N	S	N	CS	Numbers/rate
Grivell 1981	1981	Australia	Hosp	Feedback	N	N	Y	N	N	N	S	N	BA	Numbers/rate
Hardwick 1982	1982	Canada	PC	Funding policy and guidelines	N	N	N	N	Y	Y	M	Y	BA	Numbers/rate
Hardwick 1982	1982	Canada	PC	Funding policy and guidelines	N	N	N	N	Y	Y	M	Y	BA	Pattern
Hardwick 1982	1982	Canada	PC	Funding policy and guidelines	N	N	N	N	Y	Y	M	Y	BA	Expenditure
Larson 1999	1999	Sweden	PC	Education	N	Y	N	N	N	N	M	N	BA	Pattern
Mindemark 2009	2009	Sweden	PC	Education	N	Y	N	N	N	N	M	N	BA	Pattern
Horn 2014	2014	USA	PC	Display of cost of the tests being ordered	N	N	N	Y	N	N	M	N	ITS	Numbers/rate
Nightingale 1994	1994	UK	Hosp	Education and feedback and protocol management sys	N	Y	Y	Y	N	N	M	N	BA	Appropriateness/compliance
Rhyne 1979	1979	USA	PC	Education and guidelines	N	Y	N	N	N	Y	M	Y	BA	Numbers/rate
Rhyne 1979	1979	USA	PC	Education and guidelines	N	Y	N	N	N	Y	M	Y	BA	Appropriateness/compliance
Schectman 1991	1991	USA	PC	Educational memorandum	N	Y	N	N	N	N	S	Y	CS	Numbers/rate
Schectman 1991	1991	USA	PC	Educational memorandum	N	Y	N	N	N	N	S	Y	CS	Appropriateness/compliance
Schectman 1991	1991	USA	PC	Reminders	Y	N	N	N	N	N	S	Y	CS	Appropriateness/compliance
Schectman 1991	1991	USA	PC	Feedback and reminders	Y	N	Y	N	N	N	M	Y	CS	Appropriateness/compliance
Stuart 2002	2002	Australia	Hosp	Education and feedback and guidelines	N	Y	Y	N	N	Y	S	N	BA	Expenditure
Thomas 2006	2006	UK	PC	Feedback	N	N	Y	N	N	N	S	N	RCT	Numbers/rate
Thomas 2006	2006	UK	PC	Reminders	Y	N	N	N	N	N	S	N	RCT	Numbers/rate
Thomas 2006	2006	UK	PC	Feedback and reminders	Y	N	Y	N	N	N	M	N	RCT	Numbers/rate
Tierney 1988	1988	USA	PC	Display of computer-generated probability estimates	N	N	N	Y	N	N	S	N	RCT	Expenditure
Tomlin 2011	2011	NZ	PC	Education and feedback and guidelines	N	Y	Y	N	N	Y	M	Y	CS	Numbers/rate
Tomlin 2011	2011	NZ	PC	Education and feedback and guidelines	N	Y	Y	N	N	Y	M	Y	CS	Expenditure
Tomlin 2011	2011	NZ	PC	Education and feedback and guidelines	N	Y	Y	N	N	Y	M	Y	CS	Pattern
Toubert 2000	2000	France	Hosp	Guidelines and reminders	Y	N	N	N	N	Y	M	Y	BA	Numbers/rate
Toubert 2000	2000	France	Hosp	Guidelines and reminders	Y	N	N	N	N	Y	M	Y	BA	Appropriateness/compliance
Toubert 2000	2000	France	Hosp	Guidelines and reminders	Y	N	N	N	N	Y	M	Y	BA	Pattern
van Gend 1996	1996	The Nether	PC	Request form redesign and feedback	N	N	Y	Y	N	N	M	N	BA	Pattern
van Walraven 1998	1998	Canada	PC	Guidelines and funding policy	N	N	N	N	Y	Y	M	N	ITS	Numbers/rate
van Walraven 1998	1998	Canada	PC	Guidelines and request form redesign	N	N	N	Y	N	Y	M	N	ITS	Numbers/rate
Vidal-Trecan 2003	2003	France	Hosp	Education and guidelines and request form redesign	N	Y	N	Y	N	Y	M	Y	BA	Numbers/rate
Vidal-Trecan 2003	2003	France	Hosp	Education and guidelines and request form redesign	N	Y	N	Y	N	Y	M	Y	BA	Pattern
Willis 2013	2013	UK	Hosp	Education and guidelines	N	Y	N	N	N	Y	M	N	BA	Numbers/rate
Wong 1983	1983	USA	Hosp	Guidelines and request form redesign	N	N	N	Y	N	Y	M	N	CS	Numbers/rate
Wong 1983	1983	USA	Hosp	Guidelines and request form redesign	N	N	N	Y	N	Y	M	N	CS	Pattern

Direction	Large effect	Not chance	Notes
+	+	+	% admissions offered TFT
+	-	-	Per 1000 registered patients
+	-	NR	Per 100 encounters
-	+	NR	Per 100 encounters
+	-	NR	Per 100 encounters
+	+	NR	Per 100 encounters
+	+	NR	Per 100 encounters
-	-	NR	Per 100 encounters
+	+	+	Per 100 ED visits
+	-	NR	Per discharge
+	-	-	Proportion of TFTs ordered in accordance with the guidelines
+	+	+	Proportion of TFTs ordered in accordance with the guidelines
+	-	+	Proportion of TFTs ordered in accordance with the guidelines
+	-	+	Per patient visit
+	+	+	Per patient visit
+	NR	+	
+	+	NR	Sought to shift to TSH and cascade
+	-	NR	Per 1000 patients
+	+	NR	Sought to shift to TSH and TSH-based algorithm
+	+	+	Per outpatient visit
-	+	NR	
+	-	NR	Population-based study
+	-	NR	Population-based study
+	+	NR	Population-based study
+	-	+	Sought to shift to TSH; and reduce ordering of TT3 and FTT4 relative to TSH. Summary based on TSH/all TFTs ratios
-	-	+	Sought to shift to TSH; and reduce ordering of TT3 and FTT4 relative to TSH. Summary based on TSH/all TFTs ratios
+	-	-	Per 1000 visits
+	+	NR	% patients requiring a particular investigation according to protocol who were actually testec
+	+	+	Per 100 encounters
+	-	-	% "high" and "low" indications
+	-	+	Per patient
+	+	+	Compliance with TSH-only strategy
+	-	+	Compliance with TSH-only strategy
+	-	+	Compliance with TSH-only strategy
+	+	+	Mean cost per patient
+	-	+	Per 10000 registered patients
+	-	+	Per 10000 registered patients
+	-	+	Per 10000 registered patients
+	-	-	Charges per scheduled visit (in USA$)
+	+	+	Per year per GP
+	-	NR	
+	+	+	Sought to shift to TSH. Summary based on % TFTs TSH alone
+	+	NR	
+	+	+	% appropriate
+	+	NR	Sought to shift to TSH. Summary based on % TFTs TSH alone
+	+	NR	Sought to shift away from TT4. Summary based on FTT4:TSH ratio
+	+	+	Summary based on decrease in the proportion of TT4 and T3RU
+	-	+	Summary based on decrease in TSH utilisation
+	-	NR	Summary based on the total number of TFTs
+	-	NR	Sought to shift to TSH. Summary based on the proportion of FT3 and TSH
+	+	+	Per admission
+	+	NR	
+	+	NR	Sought to decrease ordering of "complete" thyroid panel to more selective use of individual tests
